# Estrogen receptors α, β and GPER in the CNS and trigeminal system - molecular and functional aspects

**DOI:** 10.1186/s10194-020-01197-0

**Published:** 2020-11-10

**Authors:** Karin Warfvinge, Diana N. Krause, Aida Maddahi, Jacob C. A. Edvinsson, Lars Edvinsson, Kristian A. Haanes

**Affiliations:** 1 Department of Clinical Experimental Research, Glostrup Research Institute, Rigshospitalet, Glostrup, Denmark; 2 Division of Experimental Vascular Research, Department of Clinical Sciences, Lund University Hospital, Lund, Sweden; 3 Department of Pharmacology, School of Medicine, University of California at Irvine, Irvine, CA USA; 4 Department of Drug Design and Pharmacology, Faculty of Health and Medical Sciences, University of Copenhagen, Copenhagen, Denmark; 5 Department of Internal Medicine, Lund University Hospital, S-22185 Lund, Sweden

**Keywords:** Estrogen receptors, CGRP, PACAP, Immunohistochemistry, Myography, Brain, Trigeminal ganglion, MCA

## Abstract

**Background:**

Migraine occurs 2–3 times more often in females than in males and is in many females associated with the onset of menstruation. The steroid hormone, 17β-estradiol (estrogen, E2), exerts its effects by binding and activating several estrogen receptors (ERs). Calcitonin gene-related peptide (CGRP) has a strong position in migraine pathophysiology, and interaction with CGRP has resulted in several successful drugs for acute and prophylactic treatment of migraine, effective in all age groups and in both sexes.

**Methods:**

Immunohistochemistry was used for detection and localization of proteins, release of CGRP and PACAP investigated by ELISA and myography/perfusion arteriography was performed on rat and human arterial segments.

**Results:**

ERα was found throughout the whole brain, and in several migraine related structures. ERβ was mainly found in the hippocampus and the cerebellum. In trigeminal ganglion (TG), ERα was found in the nuclei of neurons; these neurons expressed CGRP or the CGRP receptor in the cytoplasm. G-protein ER (GPER) was observed in the cell membrane and cytoplasm in most TG neurons. We compared TG from males and females, and females expressed more ER receptors. For neuropeptide release, the only observable difference was a baseline CGRP release being higher in the pro-estrous state as compared to estrous state. In the middle cerebral artery (MCA), we observed similar dilatory ER-responses between males and females, except for vasodilatory ERβ which we observed only in female arteries.

**Conclusion:**

These data reveal significant differences in ER receptor expression between male and female rats. This contrasts to CGRP and PACAP release where we did not observe discernable difference between the sexes. Together, this points to a hypothesis where estrogen could have a modulatory role on the trigeminal neuron function in general rather than on the acute CGRP release mechanisms and vasomotor responses.

## Introduction

Migraine is a neurological disorder that affects 15% of the global population, predominates in the most active period of life and has enormous socioeconomic impact on society and family [1]. Migraine occurs 2–3 times more often in females than in males and is in many females associated with onset of menstruation [2]. This has resulted in much work aimed to unravel the role of sex hormones on migraine pathophysiology and to alleviate symptoms in females by substitution of sex hormones [3]. The steroid hormone, 17β-estradiol (estrogen, E_2_), primarily produced in the female ovaries, has hence taken a dominant role in explanations of sex difference in migraine prevalence. Estrogen exerts its effects by binding and activating several estrogen receptors (ERs). In addition to the ovaries, fat cells produce active estrogen precursors in both sexes, and this is also the major site for formation of estrogen after menopause. Estrogen is further produced in the brain and in arterial walls [4]*.* Despite the possible negative effect on migraine prevalence linked to estrogen, there are several beneficial estrogenic effects, which include anti-apoptotic, anti-inflammatory and anti-oxidant properties as well as its ability to increase cerebral blood flow [5].

The two main ER, α and β, were originally thought only to act as transcription factors and appear to be located predominantly in synapses, axons, dendrites and dendritic spines [6, 7]. In contrast to the original hypotheses of being pure modulators of transcription, both ERα and ERβ localize in many cells to the plasma membrane (5–10% of total cellular ER) and to cytoplasmic organelles including mitochondria and endoplasmic reticulum [8]. The newest addition is the G protein-coupled estrogen receptor 1 (GPER) which is localized to the endoplasmic reticulum and may contribute to normal estrogen physiology as well as pathophysiology [9]. GPER function to regulate synaptic plasticity, at least in part, by acting on synaptic proteins [10]. Not much is known about the function of this receptor in males, despite evidence that points towards possible sexual dimorphism [11].

Calcitonin gene-related peptide (CGRP) has a strong position in migraine pathophysiology [12]. Thus, monoclonal antibodies and gepants are examples of the rapidly growing body of medications designed to modify CGRP mechanisms in the trigeminovascular system [13] (TVS); these are successful drugs for acute and prophylactic treatments of migraine in all age groups and in both sexes [14]. However, new targets are needed [15].

Besides of genomic effects that induce activation of nuclear receptors, transcription and translation, estrogen has been shown to exert non-genomic acute effects on vascular tone such as vasodilatation by Ca^2+^ channel antagonistic effects that inhibits extracellular influx of Ca^+^ [16] and modulation of the Rho/Rho kinase pathway and phosphorylation of myosin light chain [17] as well as via an endothelial effect mediated via release of nitric oxide [18].

The most striking link between estrogen and migraine is that in biologically-predisposed women, migraine attacks are triggered by a decline in plasma estrogen concentrations in the late luteal (premenstrual) phase of the menstrual cycle [19, 20]. On the contrary, phases of rising estrogen levels appear to protect against migraine [3]. A key question is, however, why does estrogen affect migraine mechanisms in some but not in others? The presence of sex hormone receptors in the TVS suggests that trigeminal neurons are sensitive to variations in the levels of these hormones [21] and some of this could be linked to oxytocin [22].

We asked the questions: where are the estrogen receptors expressed in the CNS and in the TVS, using a palette of specific antibodies. Further we specifically looked at difference in the trigeminal ganglion (TG) between males and females, both in ER expression and in the CGRP release. Finally, we used the middle cerebral arteries (MCA) as a proxy for the TVS measuring functional difference between male and female response to estrogen.

## Material and methods

### Animal experiments

Adult male and female rats were anesthetized by CO_2_ inhalation followed by decapitation (*n* = 26, 250–300 g). Shortly after this, the brains (males) and the TGs (both males and females), as well as cerebral arteries were carefully dissected out and thereafter the brains were cut sagittal in the midline. The brains, the TGs and the cerebral arteries were submerged in 4% paraformaldehyde (PF) in phosphate buffered saline (PBS) buffer for 2–4 h. Subsequently, the tissues were incubated in rising concentration of 10% and 25% of sucrose in Sorensen’s phosphate buffer (pH 7.2) to ensure cryo-protection. Finally, the tissues were embedded in a gelatin medium (30% egg albumin, 3% gelatin), sectioned at 10 μm (TGs and arteries) or 12 μm (brains) thick slices and stored at − 20 °C. The method of PF fixation allows for a proper fixation up to 2–3 mm from the surface of the brain and inwards [20]. Since we examine a brain volume of 1 mm lateral to the midline (+ 0.5 − + 1.5 mm), a proper fixation of the tissue used for the immunohistochemical investigation is achieved.

The 6th edition of The Rat Brain in Stereotaxic Coordinates by Paxinos and Watson [23] and hematoxylin-eosin (HE) staining of sagittal sections spanning over 0.5 mm to 1.5 mm lateral to the midline were used to identify the different areas of our study. The methods used in the present study are technically similar to that published earlier [24].

#### Hematoxylin-eosin (HE)

**Fig. 1:**
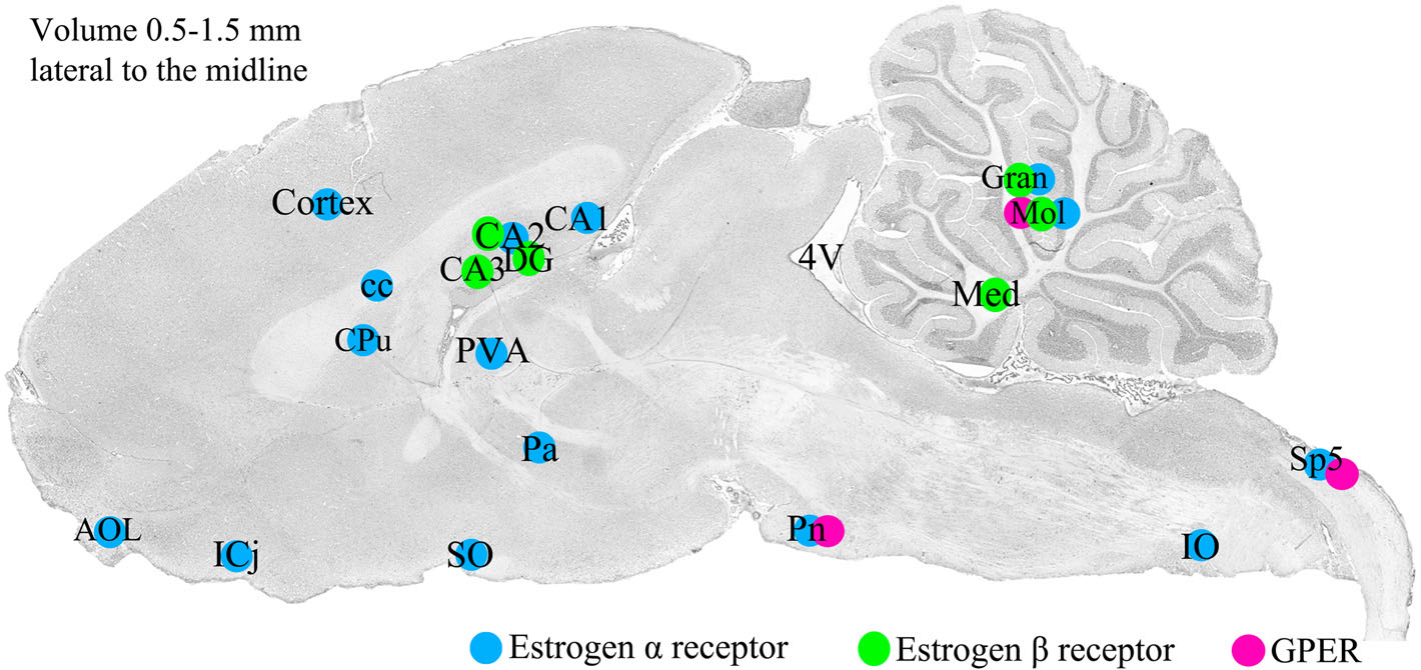
Sagittal cryo-sections stained with Hematoxylin and Eosin. The 6th edition of The Rat Brain in Stereotaxic Coordinates by Paxinos and Watson [23] was used to identify the different areas. Blue color denotes ERα receptor green color ERβ receptor and pink color GPER immunoreactivity

Cryo-sections of the whole brain, including cerebrum, cerebellum, brainstem and C_1_ spinal cord, and TGs, were stained using HE (Htx 4 min, Eosin 1 min). The staining was done in order to examine the morphology and condition of the tissue, and to identify the distance of the section of the brain from the midline. HE-staining of sagittal sections spanning over 0.5 mm to 1.5 mm lateral to the midline were used to identify the different areas subjected to the detailed study of the estrogen receptors ERα, ERβ and GPER distribution (Fig. 1).

#### Immunohistochemistry

Brain, TG and cerebral arteries sections cut in a cryostat (Microm HM500M; Thermo Scientific, Walldorf, Germany) were washed in PBS containing 0.25% Triton-X (PBS-T) for 15 min followed by application of the primary antibodies (Table 1) with incubation overnight at + 4 °C in moisturized incubation chambers. The following day, the sections were rinsed twice in PBS-T for 15 min prior to incubation with secondary antibodies (Table 1) for 1 h at room temperature. Finally, the sections were washed 2 × 15 minutes and mounted with cover glass and Vectrashield mounting medium containing 4′,6-diamidino-2-phenylindole (DAPI) (Vector Laboratories, Burlingame CA, USA). Negative controls were performed for each set by omitting the primary antibody (not shown). Any resulting immunofluorescence would suggest unspecific binding of the secondary antibodies.

**Table 1:** Details of primary and secondary antibodies

Antigen	Dilution	Species	Immunogen	Supplier
**Primary antibodies**				
CGRP (PA1–36017)	1:500	guinea pig	Synthetic human CGRP	Thermo Scientific, IL, USA
Estrogen Receptor α (H-184, sc-7207)	1:100	rabbit	Amino acids 2–185 of ER α of human origin	Santa Cruz Biotechnology, CA, USA
Estrogen Receptor α (ab75635)	1:100	rabbit	Synthetic peptide corresponding to Human Estrogen Receptor alpha (N terminal).	Abcam, Cambridge, UK
Estrogen Receptor β (PA1–311)	1:100	rabbit	Synthetic peptide corresponding to residues A(55)EPQKSPWCEARSLEH(70) of rat ER beta	Thermo Scientific, IL, USA
Estrogen Receptor β (ab288)	1:250	rabbit	Recombinant 6-His fusion protein encoding amino acids 1–153 of human ER expressed in E.coli	Abcam, Cambridge, UK
GPER (G protein-coupled estrogen receptor 1)	1:100	rabbit	(C) ETFRDKLRLYVAQK, corresponding to amino acid residues 329–342	Alomone Labs, Ltd., Jerusalem, Israel
CASPR (contactin associated protein 1)	1:100	mouse	Recombinant protein corresponding to rat Caspr	Millipore Corporation, Temecula, CA, USA.
**Secondary antibodies**				
Anti-guinea pig	1:100	Alexa 488	Thermo Scientific, IL, USA	
Anti-guinea pig	1:400	Cy3	Jackson Immunoresearch Laboratories, West Grove, PA, USA	
Anti-rabbit	1:400	Cy3	Jackson Immunoresearch Laboratories, West Grove, PA, USA	
Anti-rabbit	1:100	Cy2	Jackson Immunoresearch Laboratories, West Grove, PA, USA	
Anti-mouse	1:400	Cy3	Jackson Immunoresearch Laboratories, West Grove, PA, USA	

For double immunohistochemistry, the procedure described above was repeated two times. The first primary antibody was matched with its appropriate secondary antibody before the second round of primary and secondary antibodies were applied and finally mounted.

Quantification of ERα, ERβ and GPER immunoreactive cells was performed in male and female rats. Estimation of the prevalence ERα, ERβ and GPER in the TGs were determined by counting intermingled cells in pooled ophthalmic, maxillary and mandibular areas of each ganglion [25]. We counted cells only intermingled in fibers since our experience is that neurons close to the surface may show artefactual fluorescence. All negative and immunoreactive cells were counted in each area. The mean percentage of positive neurons (small and medium sized) in 3 slides/rat from 3 male or female rats were calculated (Table 2).

**Table 2:** Percentage of immunoreactive neurons in male and female TG

	Male	Female
ERα	32%	59%
ERβ	34%	72%
GPER	48%	65%

The sections were examined in a light and epifluorescence microscope (Nikon 80i, Tokyo, Japan) equipped with a motor table, enabling us to get images of a whole section, and with a Nikon DS-2MV camera. Finally, images were processed using Adobe Photoshop CS3 (v0.0 Adobe Systems, Mountain View, CA).

Dura mater spreads were collected from 5 additional male rats (approx. 350 g) for double immunohistochemistry. An antibody for contactin associated protein 1 (CASPR) was employed in combination with antibodies for either ERα, ERβ or GPER to elucidate their presence in myelinated dura fibers.

#### CGRP/PACAP release in males and females

Adult female and male rats (12 females and 10 males, 10–12 weeks old) were purchased from Taconic (Ejby, Denmark). All these procedures are approved by the Danish Animal Experimentation Inspectorate. The protocol is described in detail elsewhere [26, 27]. Rats were anaesthetized by CO_2_ inhalation and decapitated. The skull was cut mid-sagittal and the brain halves were carefully removed while the cranial dura was left attached to the skull, and the TG was carefully dissected out. For the buffer system, 300 μL of synthetic interstitial fluid (SIF, composition: 108 mM NaCl, 3.5 mM KCl, 3.5 mM MgSO_4_, 26 mM NaHCO_3_, NaH_2_PO_4_, 1.5 mM CaCl_2_, 9.6 mM NaGluconate, 5.6 mM glucose and 7.6 mM sucrose; pH 7.4) at + 37 °C was used. TGs were randomized, placed in Eppendorf tubes in a heating block at + 37 °C and washed. For the skull halves, these were also randomized and placed in a humid chamber above a water bath to maintain temperature at + 37 °C. 200 μL samples for measuring CGRP/PACAP content were collected from both tissues 10 min after stimuli, mixed with 50 μL enzyme immunoassay buffer (containing protease inhibitors) and stored at − 20 °C until analysis, within a week after the experiment was performed. The release of CGRP and PACAP was induced by 60 mM potassium. To maintain equal osmolality in the 60 mM K^+^ buffer, a proportional amount of Na^+^ was removed from the buffer.

The samples (100 μL for CGRP and 100 μL for PACAP) were processed using commercial EIA kits, Human CGRP ELISA KIT (SPIbio, Paris, France) to study CGRP with a detection limit of 0.7 pg/mL and specificity for rat CGRP-α/β at 120%. For PACAP release the rat PACAP ELISA Kit (LS-Bio, LS-F16956, WA, USA) with detection rate: 6.25–400 pg/mL and sensitivity: 6.25 pg/mL was used. The determination of CGRP and PACAP content was based on a standard curve which was run parallel to the experiment. The protocol was performed following the manufacturer’s instructions and the optical density was measured at 410 nm using a micro-plate photometer (Tecan, Infinite M200, software SW Magellan v.6.3, Männedorf, Switzerland).

#### Pressurized arteriography experiments on rat middle cerebral artery

Male (*n* = 6) and female (*n* = 6) rats (250–300 g) were sacrificed by CO_2_ inhalation and decapitation, thereafter the brains were removed and immediately chilled in cold bicarbonate buffer solution (see *Drugs, Chemicals and Solutions*). Middle cerebral arteries (MCAs) were carefully dissected free from adhering tissue and cut into 1–2 mm cylindrical segments. Each MCA was cannulated with glass micropipettes mounted in an arteriography (Living Systems, Burlington, VT) as described earlier [28, 29]. Bicarbonate buffer solution in the arteriography was warmed to + 37 °C and continuously bubbled with 5% CO_2_ and air, resulting in a pH of 7.4. A transmural pressure of 85 mmHg was maintained by raising reservoirs connected to the micropipettes. Luminal perfusion was adjusted to 100 μL/min (range 80–100 μL/min) by setting the two reservoirs at different heights. The mounted segment was magnified 600-fold with a microscope coupled to a digital camera (Axis, Lund, Sweden) connected to a computer. Images were captured and saved at an interval of 1/s by a specifically generated software (Mary, Nihil KB, Lund, Sweden) as well as measuring of outer vessel diameter.

After mounting and pressurizing, during a 1 h equilibration period, the vessels developed a spontaneous tone. Any segment that did not develop spontaneous tone of at least 10% compared to the initial diameter was excluded. Experimental protocols were not initiated until the vessel diameter was stable over a 15 min period. Presence of functional endothelium was assessed 30 min later by luminal application of adenosine triphosphate (ATP), 10 μM. A dilation of at least 10% of the resting diameter was considered indicative of functional endothelium. Cumulative concentration-response curves were performed by luminal or abluminal application of estrogen or estrogen receptor agonists in the concentration range 10^− 8^ to 10^− 4^ M.

Substances used were 17β-estradiol (dual ERα and ERβ receptor agonist), propyl-pyrazole-triol (PPT, ERα receptor agonist), diarypropiolnitrile (DPN, ERβ receptor agonist) or equal volumes of vehicle alone (diemithylsulfoxide (DMSO) and ethanol). The vessel segments were exposed to calcium free buffer solution at the end of each experiment to allow for recalibration of the maximum relaxant capability of the MCA segment.

#### Wire myography studies of human dura vessels

The human middle meningeal artery (MMA) samples were obtained from patients (after written approval, mean age 58 years (*n* = 6), range 39–73 years) undergoing neurosurgery at the Lund University Hospital, Sweden, for removal of a tumor. The vessels were from visually healthy region and immediately after removal immersed into cold Dulbecco’s modified Eagle’s medium (DMEM, Gibco, Invitrogen, Carlsbad, CA, USA). Upon arrival to the laboratory (about 30 min later), the vessel was carefully dissected, cut into segments (2 mm long) were mounted onto a wire myograph (Multi Myograph System—610 M, Danish Myo Technology, Aarhus, Denmark) [30] as previously described [31, 32]. Briefly, two tungsten wires with a diameter of 40 μm (depending on vessel diameter) were threaded through the lumen of the segment. One wire was attached to the stationary support driven by a micrometer, and the other was attached to an isometric force transducer. The measured isometric forces were digitized and transferred to a computer running data acquisition software (Myodaq, Danish Myo Technology). From each vessel, the resting tension–to–internal circumference ratio was determined and set at 2 mN.

The tissue baths were filled with a bicarbonate buffer solution that was gassed with 95% O_2_ and 5% CO_2_ and maintained at + 37 °C. After a 30-min equilibration period, the vessels were exposed twice to buffer solution containing 60 mmol/L KCl in order to validate contractile function. To study relaxant responses to agonists, a stable level of contraction was induced in each vessel segment with U46619 (10^− 6^ M), which gave a stable contraction. Increasing concentrations of a relaxant agonist (estrogen receptor agonist) was then added in a cumulative fashion to determine a concentration-response curve. The change in contraction measured in mN per mm vessel length was expressed as percent relaxation of the force measured in the vessel at the stable level of pre-contraction. Sigmoidal curve fitting of concentration-response data was done using the computer program GraphPad Prism (GraphPad Software, San Diego, CA). pEC_50_ denotes the negative logarithm of the molar concentration needed to elicit half the maximum relaxation response. To study the effects of antagonists/inhibitors, these drugs were added 30 min prior to pre-contraction and present in the bath for the remainder of the experiment.

#### Drugs, chemicals and solutions

Brains and other tissues were removed and placed into ice-cold bicarbonate buffer of the following composition: 118.3 mM NaCl, 25 mM NaHCO_3_, 4.7 mM KCl, 1.2 mM MgSO_4_, 1.2 mM KH_2_PO_4_, 15 mM CaCl_2_ and 11.1 mM glucose, pH 7.4.

Phenylephrine, carbachol and L-NAME (N^G^-nitro-L-arginine methyl ester hydrochloride) were dissolved in distilled water to a stock solution concentration of 10^− 2^ M. Prostaglandin F_2α_, indomethacin, the agonists 17ß-estradiol, PPT (4,4′,4″-(4-propyl-[1*H*]-pyrazole-1,3,5 -triyl)*tris*phenol), DPN (2,3-*bis* (4-hydroxyphenyl)-propionitrile), and G-1 ((±)-1-[(3aR*,4S*,9bS*)-4-(6-bromo-1, 3-benzodioxol-5-yl)-3a,4,5,9b-tetrahydro-3*H*-cyclop enta [*c*]quinolin-8-yl]-ethanone) and the antagonists ICI 182,780 (7α,17β-[9-[(4,4,5,5,5-pentafluoropent yl)sulfinyl]nonyl]estra-1,3,5(10)-triene-3,17-diol), MPP (1,3-*bis* (4-hydroxyphenyl)-4-methyl-5-[4-(2-piperidinylethoxy)phenol]-1*H*-pyrazole dihydrochloride), and G-15 ((3aS*,4R*,9bR*)-4-(6-Bromo-1,3-benz odioxol-5-yl)-3a,4,5,9b-3*H*-cyclopenta [*c*]quinolone) were dissolved in DMSO to a stock solution concentration of 10^− 2^ M. On the day of experiments the substances were further diluted in bicarbonate buffer solution to their final concentrations. All substances were obtained from Tocris Bioscience (Bristol, UK).

#### Analysis and statistics

Values are given as mean ± S.E.M. Number of experiments = n, which in the wire myograph experiments corresponds to number of vessel segments tested. In each experiment, segments were taken from a minimum of 3 animals. Statistical calculations were performed using GraphPad Prism. Data were analyzed by either paired Student’s T-test, or two-way ANOVA with repeated measures and Tukey’s post hoc test. *P* < 0.05 was considered significant.

## Results

### Estrogen receptors in the brain

**Fig. 2:**
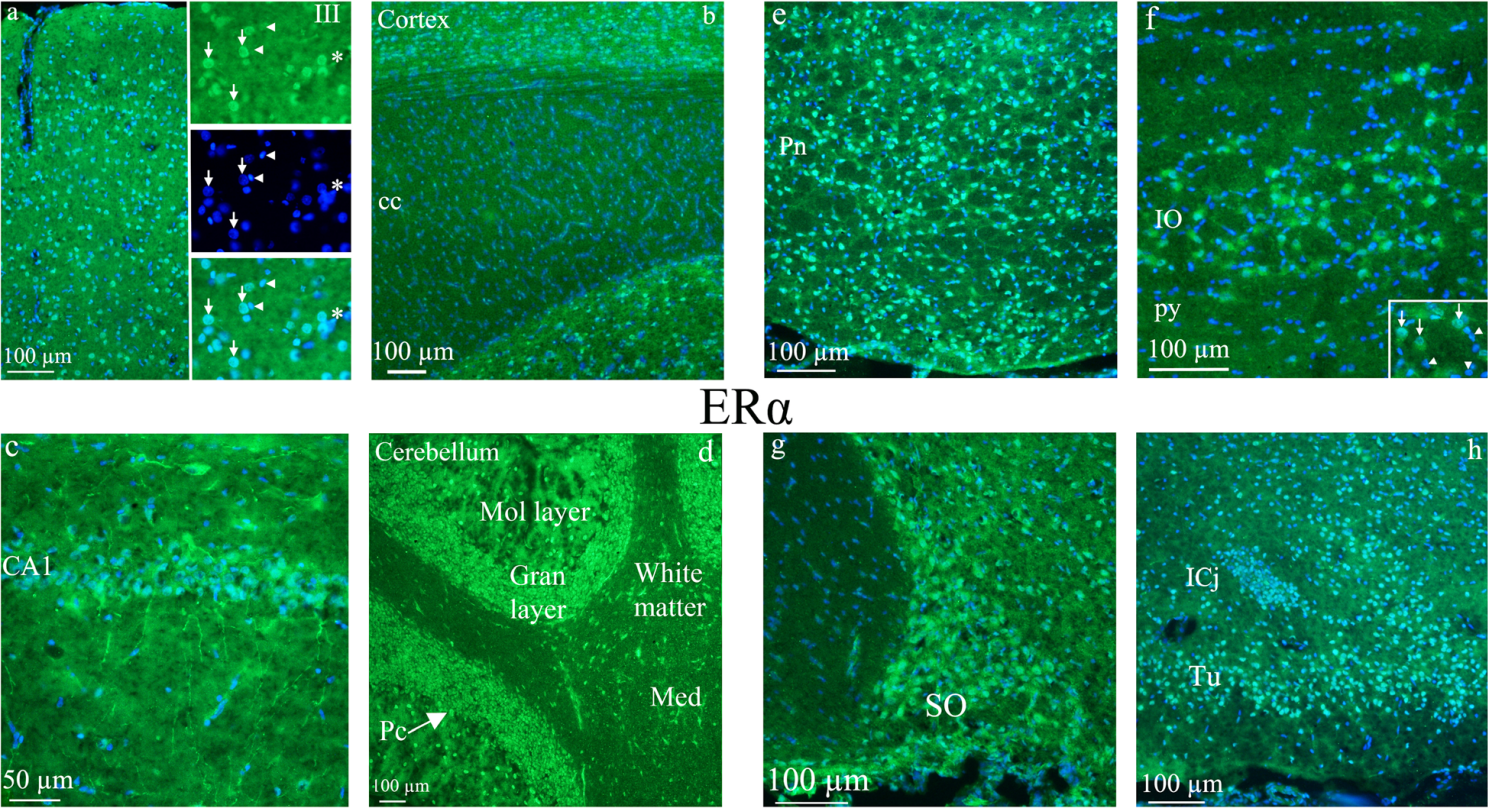
ERα receptor distribution. **a** *Cerebral occipital cortex -* all layers of the cortex contained ERα immunoreactivity, except for the outer molecular layer (layer I). In the column in the middle, from the top ERα immunohistochemistry is visualized in layer III, in the middle DAPI nuclear staining and at the bottom the merged images. Arrows point at nuclei of ERα positive neurons, arrowheads at negative glial cells and asterisk at a blood vessel. **b** In the white matter *corpus callosum* (cc), very few or no ERα expressing nuclei were found. **c** ERα receptor distribution in the *hippocampus* - in the hippocampal area CA1, ERα expression in the neuronal cytoplasm and fibers were found. **d** ERα receptor distribution in the *cerebellum* - most nuclei expressed ERα in the cerebellum. Immunoreactivity was found in the molecular (mol layer) and granular layers (gran layer), and in the Purkinje cells (Pc). In addition, expression was observed in the *Medial cerebellar nuclei* (Med). **e** and **f** ERα receptor distribution in the *Pontine nuclei* (Pn) and *Inferior olive* (IO) – Pontine nuclei is a part of the brainstem that conducts signals from the brain down to the cerebellum and medulla, and tracts that carry the sensory signals up into the thalamus. Intense neuronal nuclei expression was found in the Pn**.** The inferior olive is a structure found in the medulla oblongata and is known to coordinate signals from the spinal cord to the cerebellum to regulate motor coordination and learning. The nuclei of IO showed ERα immunoreactivity. Insert: arrows point at immunoreactive neuronal cell nuclei, arrow heads point at negative glial cells. **g** and **h** ERα receptor distribution in the *Supraoptic nucleus* (SO) and in the *Island of Calleja* (ICj) - in the supraoptic nucleus), a nucleus of neurosecretory cells (oxytocin and vasopressin producing) in the hypothalamus, intense ERα immunohistochemistry was observed. In the ventral striatum, a region of the brain which is part of the limbic system, the Island of Calleja, a group of neural granule cells, is situated. Here, the densely packed group of cells showed intense ERα immunoreactivity. Immunoreactivity was also seen in the *olfactory tubercle* (Tu)

An overview is shown in Fig. 1. We first decided to examine the estrogen receptor expression in the brain. ERα was found throughout the whole brain, including cerebrum, cerebellum, brainstem and C_1_ spinal cord (Fig. 2). The main part of this work was done on male rats. Preliminary work has now revealed that there is a rich expression of estrogen receptors also in females; however, this work will need quantitative analysis which we aim to proceed with in future studies.

For the migraine related structures, we found ERα in the supraoptic nucleus (SO) and in the paraventricular hypothalamic nucleus (Pa), and in the nuclei of neurosecretory cells (these are oxytocin and vasopressin producing) in the hypothalamus. Further, in the ventral striatum, a region of the brain, which is part of the limbic system, the Island of Calleja (ICj), a group of neural granule cells showed intense ERα immunoreactivity. Immunoreactivity was also seen in the olfactory tubercle (Tu). All layers of the cerebral cortex (Oc2; occipital cortex area 2) contained ERα immunoreactivity, except for the outer molecular layer. Pontine nuclei (Pn) is a part of the brainstem that conducts signals from the brain down to cerebellum and medulla, and tracts that carry the sensory signals up into the thalamus. Intense neuronal nuclei expression was found in Pn. The inferior olive (IO) is a structure found in the brainstem and is known to coordinate signals from the spinal cord to the cerebellum to regulate memory and learning. The nuclei of IO showed ERα immunoreactivity.

Generally, ERα expression was observed in the neuronal nuclei. In addition, ERα immunoreactivity was observed in the nucleus of glial cells in some areas, for example in the corpus callosum. Moreover, processes from the hippocampal pyramidal cells expressed the ERα. Accordingly, few or no cell nuclei were found to be ERα immunoreactive in for example fornix, optic tract inter alia (Fig. 2).

**Fig. 3:**
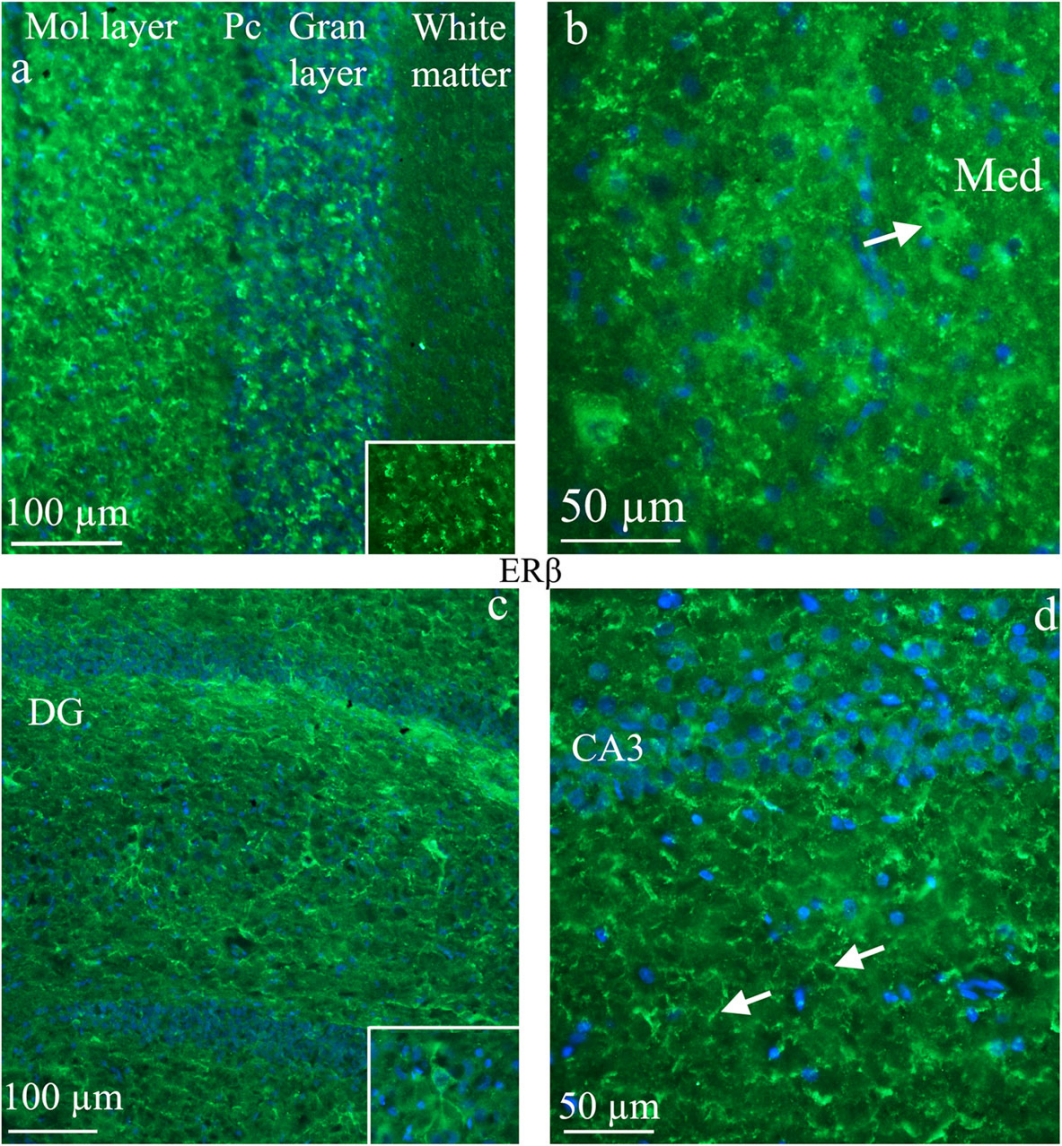
ERβ receptor distribution. **a** and **b**) ERβ receptor distribution in the *cerebellum* - the expression of ERβ was often observed in tiny loop-formations or short curvatures in areas close to the cell surface or intercellularly, reminiscent of extra cellular matrix distribution. In the cerebellum, the typical ERβ expression was found in the molecular (mol layer) and granular (gran layer) layers, but not in the Purkinje cells (Pc) and not in the white matter. Insert: staining of the granular layer. In Medial cerebellar nuclei (Med), occasional neuronal expression was observed (arrow). **c** and **d**) ERβ receptor distribution in the *hippocampus -* the dentate gyrus (DG) and CA3 are parts of hippocampus. Some ERβ positive pyramidal cells were found in DG (insert). In the other parts of hippocampus, staining resembling cell surface or extracellular matrix immunoreactivity was observed (arrows)

ERβ immunoreactivity was mainly found in the hippocampus and the cerebellum. We did not observe ERβ in structures such as thalamus and hypothalamus known to be involved in migraine pathophysiology. The expression of ERβ was often observed in tiny loop-formations or short curvatures in areas close to the cell surface or intercellularly, reminiscent of extra cellular matrix distribution. Also here, the hippocampus was the only area where neuronal cell bodies expressing ERβ could be identified. In the cerebellum, the typical ERβ expression described above was found in the molecular and granular layers, but not in the Purkinje cells and not in the white matter (Fig. 3).

**Fig. 4:**
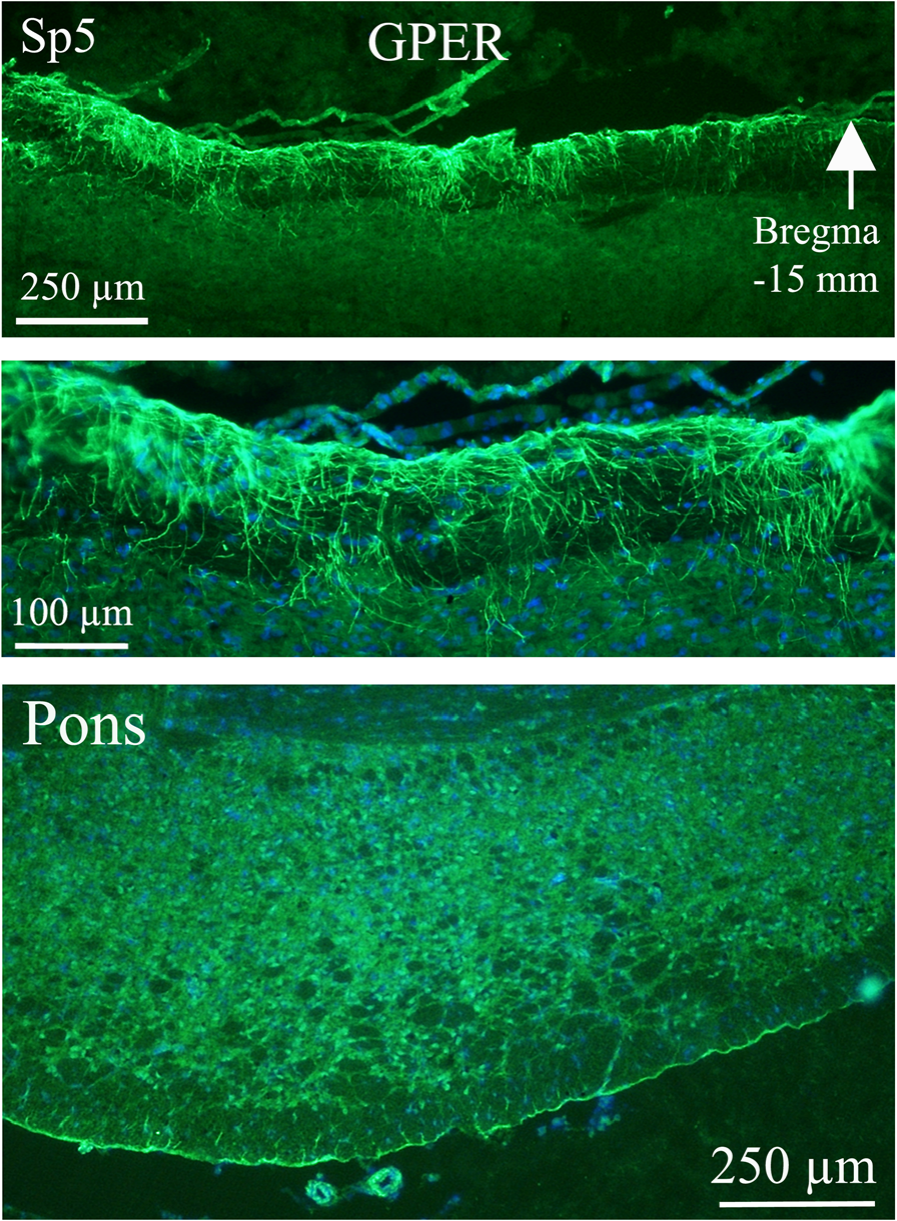
GPER receptor distribution in the Sp5 and Pontine nuclei. GPER was mainly found in the *Pontine nuclei*, cerebellar molecular layer and the spinal trigeminal tract. The *spinal trigeminal tract region* (Sp5) constitutes an essential part of the pain pathways activated in migraine attacks. We found GPER immunoreactive fibers in the Sp5 region. In Pontine nuclei the GPER immunoreactivity was mainly found in the cell nuclei, but some cytoplasmatic and fiber staining were also found

GPER was mainly found in the Pontine nuclei, cerebellar molecular layer and the spinal trigeminal tract (Sp5). In Pn the GPER immunoreactivity was found in the cell nuclei and less prominently in cytoplasmic and fiber structures. In the molecular layer of the cerebellum fiber structures were immunoreactive. We found GPER immunoreactive fibers in the Sp5 region, which constitutes an essential part of the pain pathways activated in migraine attacks (Fig. 4).

### Estrogen receptors in the trigeminal ganglion (TG)

**Fig. 5:**
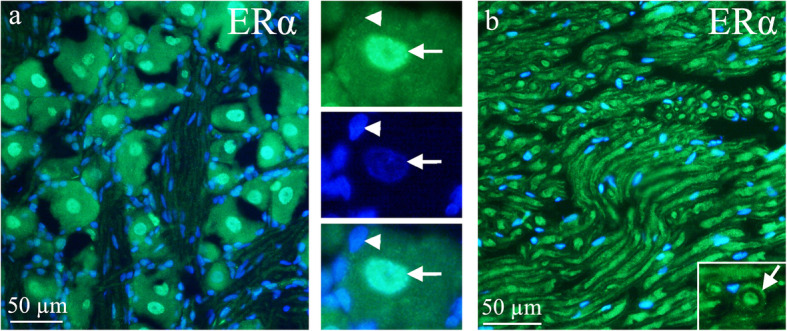
ERα receptor distribution in the TG. **a** The neuronal nuclei of the TG express ERα receptor. The montage discloses the neuronal nuclei staining, arrow heads point at a negative nucleus of the glial cells, and arrows at a positive neuronal nucleus. **b** In the trigeminal nerve, immunoreactivity was found in the nerve fibers. Insert: Perpendicular cut nerve fibers displays an inner immunoreactive fiber (arrow). Outside of the fiber, the myelin sheet showed no immunoreactivity

**Fig. 6:**
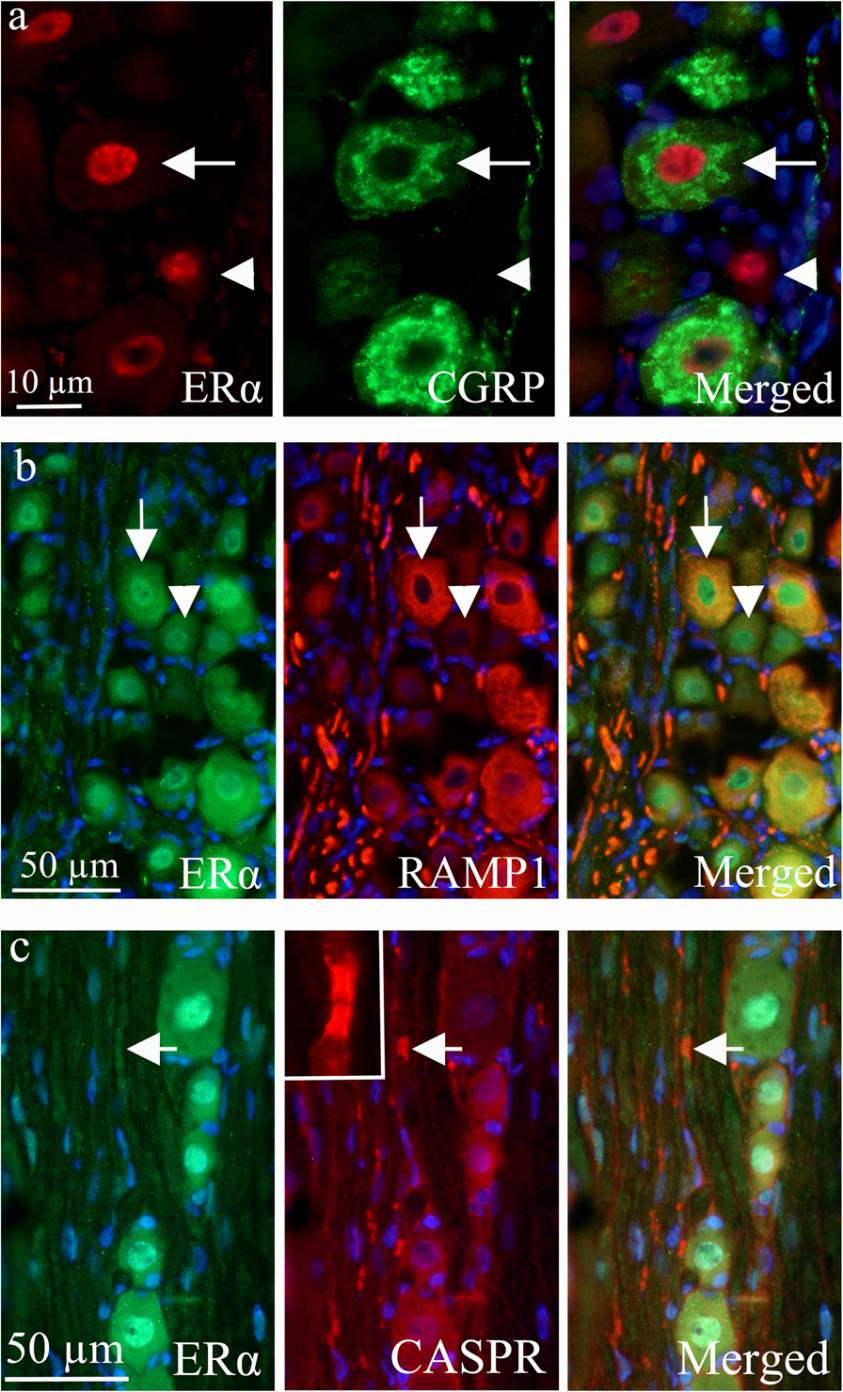
ERα receptor distribution in the TG. **a** *Double immunohistochemistry of ERα and CGRP.* To the left, the image shows ERα receptor expression in the nucleus of neurons (red). Arrow and arrowhead point at ERα immunoreactive neuronal nuclei. CGRP immunoreactive cells are shown in the middle panel (green). CGRP immunoreactivity was observed in the cytoplasm in a Golgi apparatus pattern. Arrow points at a CGRP immunoreactive neuron and arrowhead at a negative neuron. To the right, the merged image is shown. Arrow points at a neuron positive for both ERα and CGRP, but with immunoreactivity in different compartments of the cell. Arrowhead points at a ERα positive, but CGRP negative cells. **b** *Double immunohistochemistry of ERα and RAMP1.* Double immunohistochemistry with ERα (nuclei staining, arrow and arrowhead) and RAMP1 (mainly expression in medium sized cells and thick fibers, arrow points at a RAMP1 positive and arrowhead at a RAMP1 negative cell) showed that some cells expressed both ERα and RAMP1 (arrow). Fibers expressed RAMP1, but only weak ERα expression. **c** *Double immunohistochemistry of ERα and CASPR.* Double immunoreactivity of the paranodal marker contactin-associated protein 1 (CASPR) in the paranodal areas and ERα was found (arrow). Insert: paranodal area of the Ranviers node visualized by CASPR antibodies

ERα was found in the nuclei of most TG neurons, in the thicker neuronal fibers and in most of the satellite glial cells (SGC) of the TG (Fig. 5). Double immunohistochemistry with ERα (nuclei staining) and CGRP (cytoplasmic staining) showed that the ERα is expressed in cells that co-express CGRP (Fig. 6a). Double immunohistochemistry with ERα (nuclei staining) and RAMP1 (mainly expressed in medium sized cells and thick fibers, consistent with myelinated Aδ sensory fibers [33]), revealed that some cells expressed both ERα and RAMP1 (Fig. 6b). We have recently demonstrated the expression of the paranodal marker contactin-associated protein 1 (CASPR) in the paranodal areas of the different myelinated fibers inhabiting the TG and the dura mater [33]. We found ERα in the fibers that also expressed CASPR (paranodal region flanking the of nodes of Ranvier) (Fig. 6c).

**Fig. 7:**
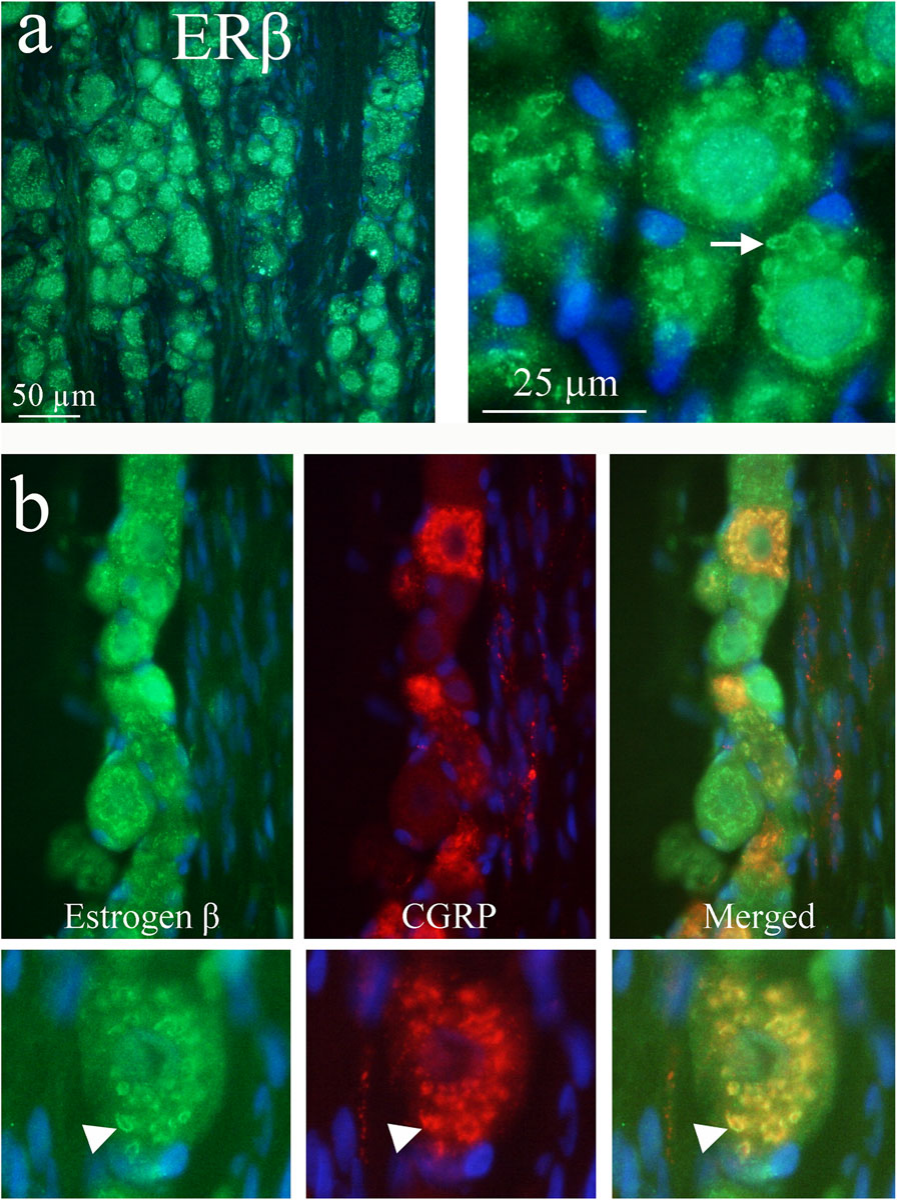
ERβ receptor distribution in the TG. **a** ERβ expression is found in the cytoplasm of most neurons. In the higher magnification to the right, arrow points at an ERβ immunoreactive neuron. **b** Double immunohistochemistry with ERβ and CGRP antibodies revealed in higher magnification that both ERβ and CGRP expression is found in the same organelle, possibly the Golgi apparatus (arrow heads)

**Fig. 8:**
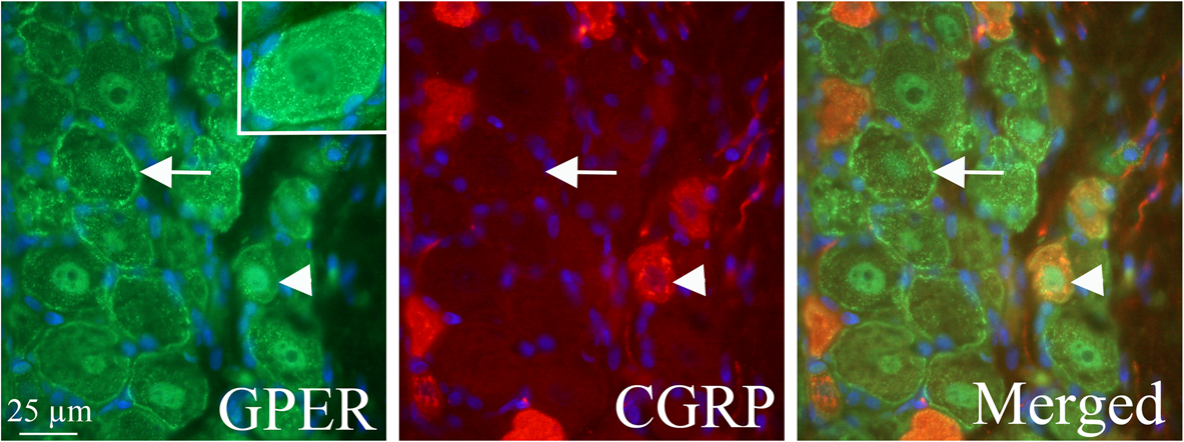
GPER and CGRP distribution in the TG. Expression of GPER was observed in the cell membrane and cytoplasm in most neurons (arrow and arrowhead). Double immunohistochemistry with GPER and CGRP showed that most CGRP positive cells (arrowhead) also expressed GPER in the cytoplasm. Insert: higher magnification of a GPER positive cell, showing immunoreactivity in the cytoplasm and cell membrane

**Fig. 9:**
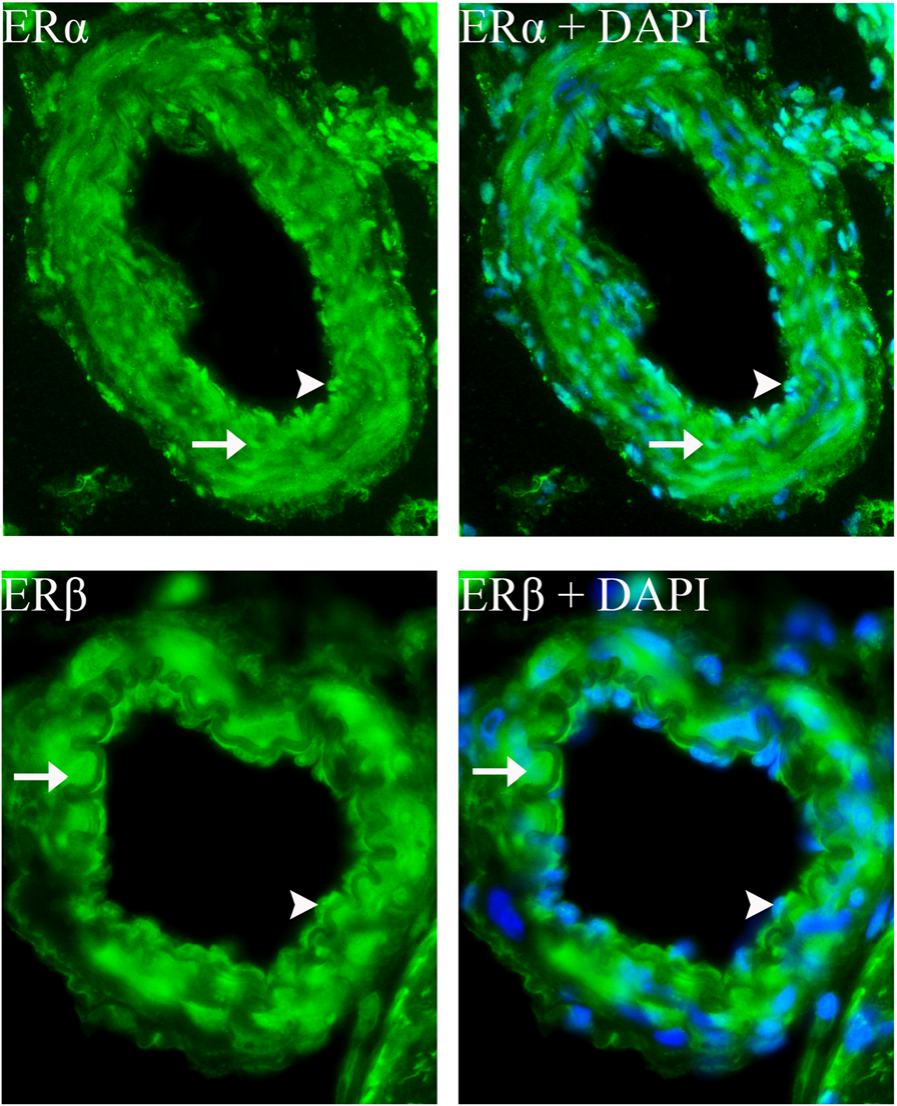
ERα and ERβ distribution in cerebral arteries. ERα and ERβ immunohistochemistry of cerebral arteries revealed that both ERα and ERβ expression was found in the smooth muscle cells (arrow) and the endothelial cells (arrowhead)

ERβ was found in the cytoplasm of trigeminal neurons. The staining pattern resembles that of the Golgi apparatus (Fig. 7a and b). Furthermore, using ERβ and CGRP antibodies it was shown that ERβ and CGRP appeared to be expressed in the same organelle (Golgi apparatus) (Fig. 7b). Expression of GPER was observed in the cell membrane and cytoplasm in most neurons (Fig. 8). Double immunohistochemistry with GPER and CGRP showed that most CGRP positive cells also expressed GPER. ERα and ERβ immunohistochemistry of cerebral arteries revealed that both ERα and ERβ expression was found in the smooth muscle cells and the endothelial cells (Fig. 9). Spread preparations revealed CASPR positive Aδ-fibers in the dura mater. However, no immunopositivity for ERα, ERβ or GPER was detected in these dura fibers (data not shown).

### Comparison of estrogen receptors in the trigeminal ganglion

**Fig. 10:**
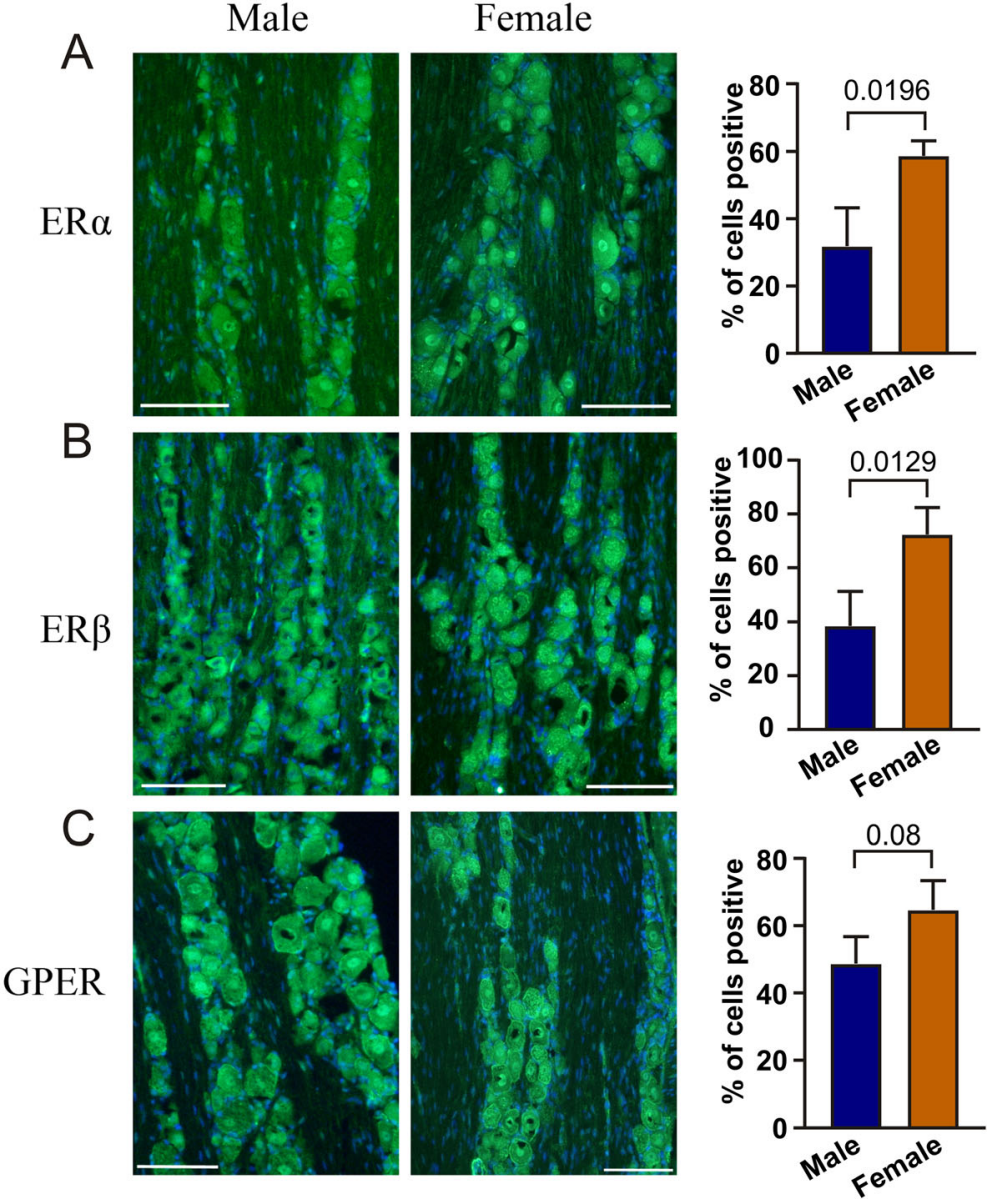
Comparison of the expression of estrogen receptors in males and females. The numbers of immunoreactive neurons in male and female TG were calculated in more than 450 neurons. The data show the distribution in males and females of ERα, ERβ and GPER. Data are shown as mean ± SEM or their individual data points with pairing, and with *p* values obtained with Student’s T-test being depicted in the graph

We then compared the trigeminal ganglion from males and females. The numbers of immunoreactive neurons in male and female TG were calculated and more than 450 neurons were counted in each group (Fig. 10). 32% of the male and 59% of the female neurons expressed ERα (*P* = 0.0196, Fig. 10a), 34% of the male and 72% of the female neurons expressed ERβ, (*P* = 0.0129, Fig. 10b). 48% of the male and 65% of the female neurons expressed GPER (*P* = 0.08, Fig. 10c).

### Release of CGRP and PACAP from dura mater and TG in males and females

**Fig. 11:**
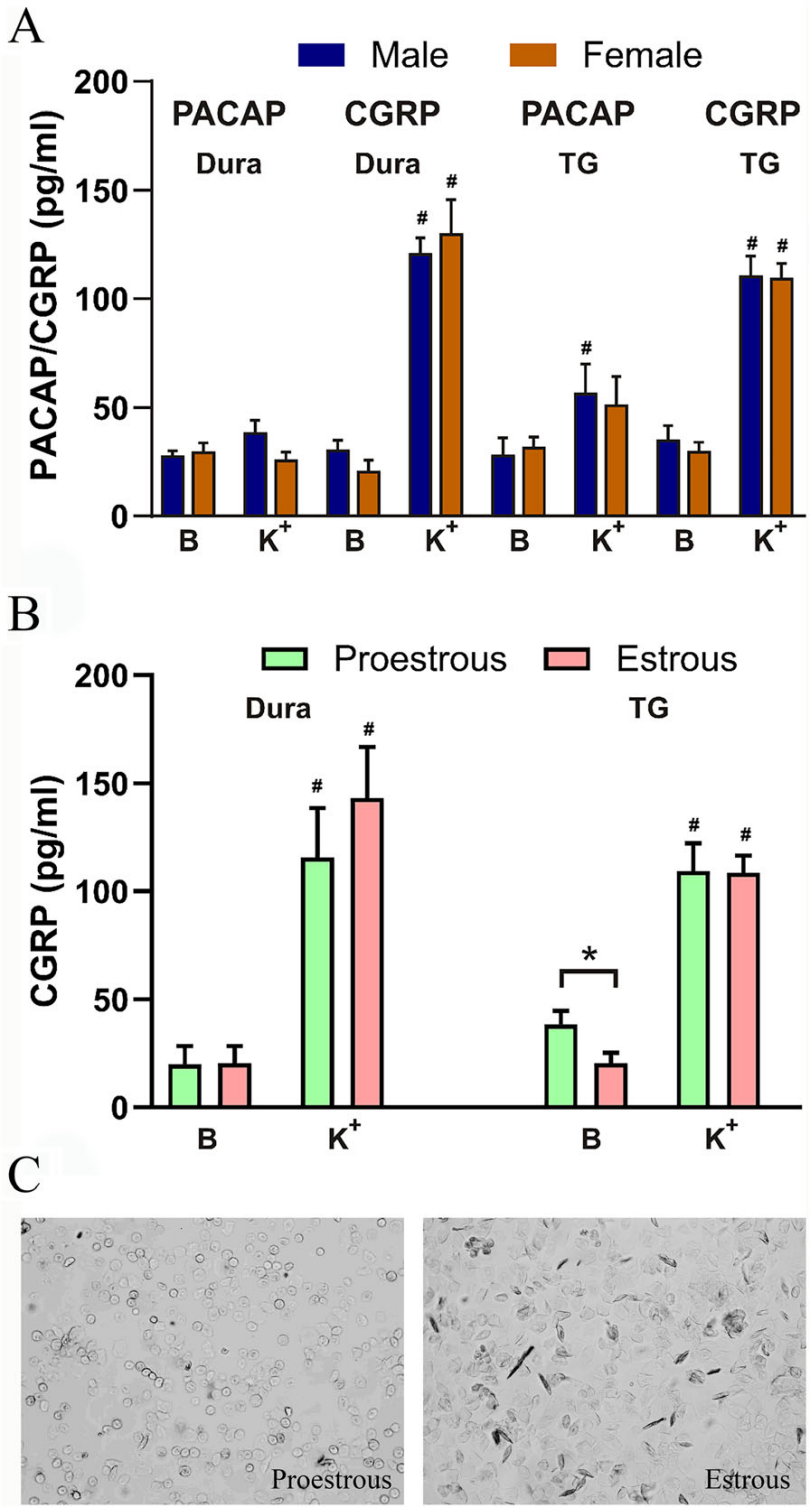
Comparison of in situ CGRP and PACAP release from the dura and trigeminal ganglion. **a** The addition of 60 mM KCl (K+) caused no PACAP release from the dura but a significant release was seen from the TG in males (*n* = 12) but not in females (*N* = 10). CGRP release was much stronger both from the dura and the TG. **b** there were no significant differences in the CGRP releases in the dura or TG, however a significant lower baseline was observed in females in estrous. **c** Representative samples of the vaginal smears from the female rats, used to determine the cycle-stage. Data are shown as mean ± SEM or their individual data points with pairing, and with *p* values obtained with Student’s T-test being depicted in the graph. * = *p* < 0.05 between groups, and # = *p <* 0.05 from baseline

To investigate a potential effect of endogenous estrogen we started out comparing CGRP release from males and females. For CGRP release (by exposure to potassium 60 mM) from the dura mater and the TG we observed a clear and significant release of CGRP but there were not any differences between sexes (Fig. 11a). There was no significant PACAP release from the dura mater from either sex. For the TG we observed significant release of PACAP from male TG (*P* = 0.0457, *n* = 12), but only a tendency in the female group (*P* = 0.1355, *n* = 10). For the CGRP release there were no differences between the sexes, and there was a strong release from both the dura mater and the TG preparations (*P* < 0.001 for both). We further separated the data from the females into two groups, based on the state of their estrous cycle (Fig. 11b). Represented photomicrographs from the vaginal smears are shown in Fig. 11c. The only observable difference was the baseline CGRP release which was higher in rats which were in the pro-estrous state as compared to the estrous state (*P* = 0.0475).

### Comparison of pressurized arteriography experiments on rat middle cerebral artery

**Fig. 12:**
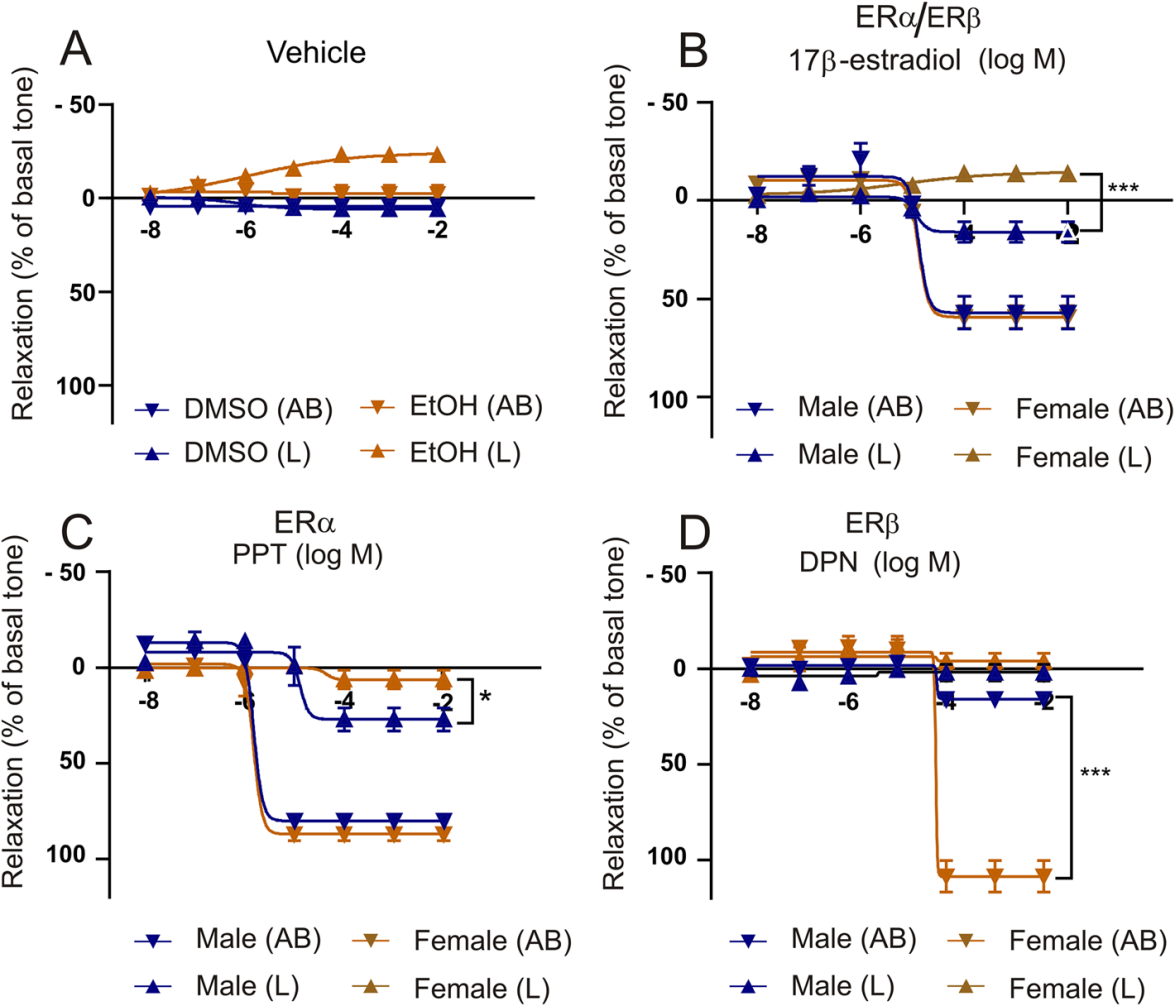
Myograph response to ERα and ERβ ligands on the male and female MCA. Isolated segments of rat middle cerebral artery (MCA), in the myograph. The data are shown as dilation from the myogenic tone. Cumulative concentration-response curves are shown for vehicle (**a**), 17β-estrogen (**b**), PPT (**c**) or DPN (**d**) added luminally (L) or abluminally (AB). Data points represent means ± SEM. Significance was determined by two-way ANOVA, *n* = 6. *p < 0.05, **p < 0.005, *** *p* < 0.001

The pressurized arteriography experiments were carried out on male and female middle cerebral arteries (MCA). After a resting period in the tissue bath the vessels attained a stable tone and tests with ATP elicited a relaxation indicative of functional endothelium [34]. The vehicles used (low dose of EtOH or DMSO) did not elicit any marked dilatory effects on the tone in concentrations equivalent to the doses used in the arteriography experiments (Fig. 12a). GPER was not tested as it has been shown not to contribute significantly to relaxant responses in the MCA [35].

The endogenous ligand for the two estrogen receptors (ERα and ERβ), estradiol (17-β-estradiol), resulted in a marked vasodilation of the MCA when given abluminal in both male 57 ± 17% and female 59 ± 13% experiments (*P* < 0.05, Fig. 12b). The luminal max vasodilation was much lower (male 16 ± 12% and female 13 ± 4%; *P <* 0.05). There were no differences between male and female.

We went further in order to examine involved receptor subtypes. Concentration-dependent responses to propylpyrazoletriol (PPT), a specific agonist for ERα, gave a strong relaxation of MCA when given abluminal in both male 79 ± 4% and female 85 ± 6% with no significant difference (Fig. 12c). For the luminal application there was a minor significant difference in the dilation (6 ± 5% for male and for female 26 ± 6%, *P <* 0.05). When given luminal only a small relaxation was seen in both male 16 ± 7% and female 6 ± 8% rat MCAs. For the ERβ, we performed concentration-dependent responses to diarylpropionitrile (DPN). It showed no or very little relaxation in males 16 ± 7%, but a large dilation in females 109 ± 17% (*P <* 0.01), when given abluminal (Fig. 12d). When given luminal, there was no relaxation observed for neither males (2 ± 9%) nor females (− 4 ± 7%). Hence, there appears to be vasodilatory ERβ only in female arteries. Immunohistochemistry revealed the presence of ERα and ERβ in the vascular smooth muscle cells (Fig. 9).

### Wire myography on human middle meningeal arteries

**Fig. 13:**
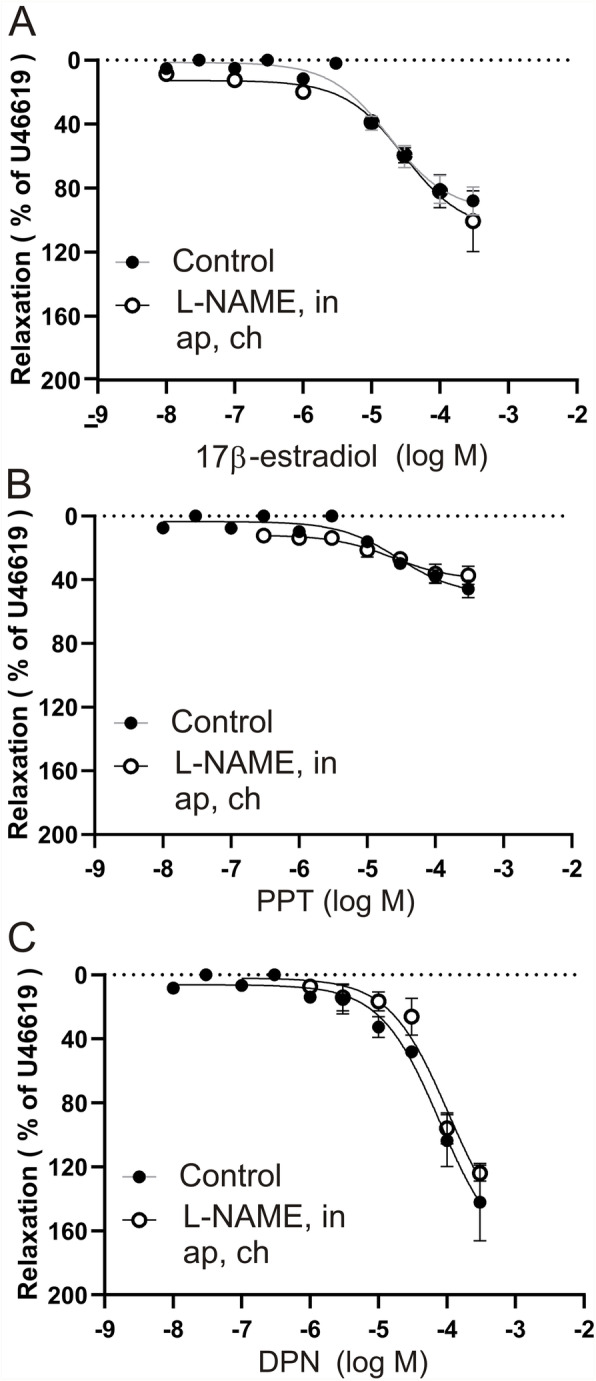
Myograph response to ERα and ERβ ligands on the human female dural vessel. Isolated segments of human female dural artery in the wire myograph. The data are shown as dilation from a preconstriction of U46619. To inhibit the endothelium L-NAME, indomethacin, apamin and charybdotoxin, where added. Cumulative concentration-response curves are shown for 17β-estrogen (**a**), PPT (**b**) or DPN (**c**). Data points represent means ± SEM. Significance was determined by two-way ANOVA, *n* = 6. *p < 0.05, **p < 0.005, *** *p* < 0.001

Since the human middle meningeal artery (MMA; a dural artery) is much larger, we opted for the wire myograph. It must be noted that in this myograph both luminal and abluminal sides are exposed to the agonists. We had arteries from 6 female patients (age avg. 58 years, range 39–73 years of age) which was found the most relevant for the current study. In addition, we blocked the endothelium responses with L-NAME, indomethacin, apamin and charybdotoxin [36, 37]. For the human MMA we observed a strong dilation (Fig. 13a) in response to 17-β-estradiol (88 ± 17%), which was unaffected by blocking the endothelium derived factors (101 ± 32%). The specific ERα agonist, PPT (Fig. 13b), showed a modest dilation of (46 ± 12%), which was not affected by blocking the endothelium (37 ± 10%). In the human arteries, it appears that ERβ (Fig. 13c) is the main receptor causing the dilation (124 ± 8%). The response is unaffected by blocking the endothelium (142 ± 42%) when tested in the presence of L-NAME, indomethacin, apamin and charybdotoxin (blocking NOS, prostacyclin and endothelium derived hyperpolarizing factor), suggesting a receptor linked to the vascular smooth muscle cells.

## Discussion

This study is the first to comprehensively analyze various aspects of the estrogen receptors in relation to migraine related structures in the brain and in the TVS. We revealed a wide distribution of estrogen receptors in the CNS and in TVS. The data showed significantly more expression of ERα and ERβ in the female trigeminal ganglion compared to males. The data indicate that there are minor differences in the level of induced CGRP and PACAP release between males and females. The ER receptor agonists revealed dilation both to estrogen and to more specific agonists, with some minor difference related to ERα and a larger significant difference in the dilation in response to the ERβ agonist. This suggests that the effect of estrogen might be linked both to neuronal modulation and to direct vascular effects in intracranial vessels.

In addition to the well-known responses to estrogens in the female reproductive system, it can act in the brain to regulate a wide range of behaviors and physiological functions in both sexes [38]. Traditionally, the actions of circulating estrogen is believed to be mediated mainly by binding to two specific receptors, ERα and ERβ, which recognize and activate gene transcription through binding to genomic elements [38]. In this study we demonstrate using immunohistochemistry ERα, ERβ and GPER expression in the rat brain and TG. We show using two different ERα antibodies that ERα is mainly expressed in the cell nucleus. By using two different ERβ antibodies we demonstrate that ERβ is mainly observed in tiny loop-formations or short curvatures in areas close to the cell surface in the CNS, but in TG the ERβ expression was observed in the cytoplasm, resembling the Golgi apparatus. GPER was expressed in a few areas in the brain, e.g. the Pontine nuclei and Sp5 (trigeminal nucleus caudalis), but in the TG almost all neurons expressed GPER on the cell surface and in the cytoplasm. In general, the data suggest that circulating estrogen might have dynamic influences on the function of the TVS and in the CNS.

Previous work, reviewed by Rossetti and coworkers [39], have shown that many cells with ERα expression are found in the bed nucleus of the stria terminalis (BST), amygdala, preoptic area and various other hypothalamic nuclei. High levels of expression are seen in olfactory regions, midbrain and cerebellum. Similar to ERα, ERβ is seen in the BST, amygdala, the trigeminal nuclei, the preoptic region, and other hypothalamic nuclei. In addition, ERβ is observed in some regions with low or no ERα, e.g. supraoptic area and paraventricular nucleus. GPER is highly expressed in the olfactory bulbs, hypothalamus, cerebral occipital cortex, hippocampus and cerebellum [39]. In the present study, we observed ERα expression throughout the whole brain, including cerebrum, cerebellum, brainstem and C_1_ spinal cord. ERβ was mainly found in the hippocampus and the cerebellum, and GPER mainly in the Pontine nuclei and Sp5. From our immunohistochemical work we agree with previous scattered studies and add a more global picture. The study includes all three estrogen receptors and add the focus to areas involved in different primary headache syndromes, putatively adding aspects to the gender imbalance in migraine prevalence (Fig. 1).

Many studies clearly indicate that ovarian hormones can alter neurotransmitter systems that play an important role in the pathogenesis of migraine headache [40]. Female sex hormones have long been considered to play a role in migraine [41, 42]. The different stages in a woman’s life where changes in female hormone levels occur (such as puberty, pregnancy, and menopause) are commonly coupled with concurrent changes in migraine frequency and severity [43, 44]. The beginning of sex hormone secretion and ending with loss of sex hormone sensitivity could frame the migraine process that is driven by sex hormones [45]. In support, we demonstrate in the present study that the numbers of ERα and ERβ expressing cells are significantly higher in female TG compared to male TG (Fig. 10).

Estrogen receptors were expressed in the same neurons as both CGRP and CGRP receptors (Fig. 6). This strengthens the hypothesis that sex hormone may modulate the CGRP system and be a key player in migraine pathophysiology [12]. For example, Wang observed that CGRP in the PAG can be modified by sex hormones [46]. Furthermore, we saw no differences in the CGRP release between males and females, this is supported by Cetinkaya et al. [47] who observed that 17β-estradiol added acutely had no effects in female peripheral terminals of meningeal trigeminal nerve or from the TG samples (compared to their controls). They did observe a minor difference in males, which might warrant future attention. Interestingly, the only difference we observed (Fig. 11) was a lower baseline in rats that are in the estrous part of the cycle. We can only speculate that CGRP might have been released during the drop in estrogen, leaving lower baseline levels. This would match the current view/hypothesis that it is the drop of estrogen that triggers migraine attack onset. PACAP release has been recently studied in detail [26], and the present study adds to the conclusion on the absence of any major difference between males and females.

Ovarian hormones passively diffuse through the blood-brain barrier [48]. Several regions in the brain, such as cortex, thalamus, amygdala, hypothalamus, pons, cerebellar deep nuclei, vestibular nucleus and Sp5, are thought to be involved in migraine based on human imaging studies and known pain pathways [49]. Migraine is commonly triggered during decline in estrogen levels before and during menstruation. Many studies have now shown that women experience an unequal amount of pain and, in addition, estrogen may be an influential factor [50, 51]. Substantial clinical evidence suggests that changes in ovarian hormones affect migraine headache [40]. The current data suggest that activation of estrogen receptors in the TG could be linked to migraine pathology. Females do not only have more cells with estrogen receptors, but appear to have stronger responses as well, using the MCA as a proxy of TVS activation. Preliminary evidence would suggest that a” threshold effect” may be the most relevant mechanism through which ovarian hormones modulate migraine headache [40]. It is very interesting that ERα is expressed in the myelinated fibers (Fig. 5), and does exist in the nodes of Ranvier (Fig. 6), which we have previously hypothesized could be a key region for modulation of migraine pain [33].

In myograph work we observed that the relaxant responses to the estrogen receptor agonists were unaffected by administration of a cocktail of endothelium relaxing factor blockers. This suggested that the main part of the estrogen receptor agonists act via the vascular smooth muscle. In support, studies with the perfusion system (selective administration of the agonists either luminal or abluminal) showed that the ER agonists had large relaxant effects given abluminal and only minor when given luminally. The abluminal application of the ERα agonist PPT showed the strongest effect followed by the mixed agonist 17-β-estradiol and less effect by DPN (ERβ) agonist in males (Fig. 12). These findings are in agreement with results from Patkar et al. [35]. Interestingly, in females DPN gave a significantly larger dilatory response than what was seen in males. When applied luminal, the responses were generally weak. Thus, the findings suggest that the ERα receptor predominates, with an additional effect of ERβ in the female MCA. This is similar to what has been shown for the rat tail artery [52]. We have similar observation for the human MMA experiments (Fig. 13), although with a smaller contribution of ERα, compared to the rat MCA data. Further, applying inhibitors of endothelium derived relaxing factors, showed that the effects are direct on the vascular smooth muscle cells, which confirms previous rat studies [35]. The immunohistochemical experiments of the ER expression agrees with the functional data, the ER expression is most obvious in the vascular smooth muscle cell layer (Fig. 9).

## Conclusion

Clearly there are numerous expression sites for estrogen receptors both in CNS and in the TVS system, all of these sites share a relation to migraine pathophysiology. We did not find a significant difference in the CGRP or PACAP release in males and females. The most striking difference is in the amount of ERα and ERβ positive cells in the female TG as compared to males. We further show that the female MCA response to ERβ in a stronger fashion that for the male counterpart while the ERα responses were similar. Together, this points to a hypothesis where estrogen could have a modulatory role on the trigeminal neuron function in general, as well as on intracranial vasodilation, rather than on the acute CGRP release mechanisms.

## Data Availability

The datasets generated during and/or analyzed during the current study are available from the corresponding author on reasonable request.

## Abbreviations

AOL: Anterior olfactory nucleus
CA1: Field CA1 of the hippocampus
CA3: Field CA2 of the hippocampus
CA3: Field CA3 of the hippocampus
Cb: Cerebellum
Cc: Corpus callosum
CPu: Striatum
DG: Dentate gyrus
Gran layer: Cerebellar granular layer
GPER: G-protein Coupled Estrogen Receptor
ICj: Islands of Calleja
IO: Inferior olive
Med: Medial cerebellar nu
Mol layer: Cerebellar molecular layer
PVA: Paraventricular thalamic nu
Pa: Paraventricular hypothalamic nu
Pn: Pontine nuclei
SO: Supraoptic nu
Sp5: Spinal trigeminal nu
TG: Trigeminal Ganglion
